# Results from a first-in-human study of dersimelagon, an investigational oral selective MC1R agonist

**DOI:** 10.1007/s00228-023-03476-6

**Published:** 2023-04-15

**Authors:** Akihito Ogasawara, Kei Ogawa, Ryosuke Ide, Yuka Ikenaga, Chie Fukunaga, Satoshi Nakayama, Minoru Tsuda

**Affiliations:** 1 Mitsubishi Tanabe Pharma Corporation, 1-1-1, Marunouchi Chiyoda-ku, Tokyo, 100-8205 Japan; 2 Mitsubishi Tanabe Pharma Corporation, Kamoshida-cho, Aoba-ku, Yokohama, Kanagawa 227-0033 Japan

**Keywords:** Erythropoietic protoporphyria, Dersimelagon, First-in human, Melanin density, X-linked protoporphyria

## Abstract

**Purpose:**

To describe outcomes from the first-in-human study of dersimelagon, an investigational oral selective MC1R agonist, under development for the treatment of erythropoietic protoporphyria (EPP) and X-linked protoporphyria (XLP).

**Methods:**

In this double-blind, placebo-controlled phase 1 study, the safety, tolerability, pharmacokinetics, and pharmacodynamics of single and multiple ascending oral doses of dersimelagon in healthy participants were evaluated.

**Results:**

Dersimelagon was generally well tolerated in healthy participants, with the most common TEAEs being lentigo (52.8%) and skin hyperpigmentation (50.0%) after multiple doses. Systemic exposure to dersimelagon in plasma (based on AUC_0-∞_ and C_max_) increased in a slightly more than dose-proportional manner over the 1- to 600-mg single-dose range. Following multiple doses, dersimelagon was rapidly absorbed (median T_max_ ranging from 4 to 5 h postdose on days 1 and 14). Mean t_1/2_ ranged from 10.56 to 18.97 h on day 14, and the steady state of plasma concentration was generally reached by 5 days of multiple dosing. There were no observable effects of age or race on the PK profile of dersimelagon or its metabolite dersimelagon glucuronide. No treatment-related effects on melanin density (MD) were observed following single doses of dersimelagon; however, after multiple doses, increases in MD were observed in participants receiving 150 and 300 mg dersimelagon.

**Conclusion:**

Our study results indicate that dersimelagon is generally well tolerated and demonstrates a generally consistent PK profile across diverse subgroups. Treatment-related increases in MD warrant further investigation in a larger study population and in patients with EPP and XLP.

**Trial registration:**

A Study to Investigate the Safety, Tolerability and Pharmacokinetics of MT-7117 in Healthy Subjects, NCT02834442, https://clinicaltrials.gov/ct2/show/NCT02834442, registration began July 2016.

**Supplementary Information:**

The online version contains supplementary material available at 10.1007/s00228-023-03476-6.

## Introduction

The porphyrias are a heterogeneous group of rare inherited metabolic disorders caused by enzymatic deficiencies of the heme biosynthetic pathway [1–3]. Erythropoietic porphyrias, which include erythropoietic protoporphyria (EPP) and X-linked protoporphyria (XLP), result from mutations of the ferrochelatase *(FECH)* gene in EPP and mutations in the aminolevulinic acid synthase-2 *(ALAS2)* gene in XLP [1–3]. EPP is the most frequent type of porphyria in children and the third most common porphyria overall, with prevalence estimates ranging from 1 in 75,000 in the Netherlands to 1 in 200,000 in the United Kingdom (UK) [1]. Both EPP and XLP are characterized by accumulation of protoporphyrin in blood, erythrocytes, and tissues, leading to cutaneous photosensitivity manifesting as burning, swelling, itching, erythema, and severe pain in sun-exposed areas [1–3].

Current approaches to the management of EPP and XLP are primarily limited to preventive measures, including sun avoidance and use of protective clothing and sunscreens, posing a substantial burden to patients by requiring significant lifestyle adjustments [1–3]. In addition, treatment options have been limited to mainly symptomatic management, while curative approaches have consisted of hematopoietic stem cell transplant for patients with EPP who have severe progressive disease and are at high risk for complications and mortality. Currently, afamelanotide, a melanocortin 1 receptor (MC1R) agonist, is the only approved pharmacological intervention indicated for use in patients with EPP [1, 4]. Afamelanotide is a subcutaneous implant and must be administered by a healthcare professional, requiring patients to commute to healthcare facilities [4]. There remains a high unmet medical need for new safe and effective treatments, and the development of a well-tolerated oral treatment for EPP and XLP may address this unmet need.

Dersimelagon, a novel synthetic orally administered non-peptide small molecule that acts as a selective agonist for MC1R, is currently being investigated for the prevention of phototoxicity in patients with EPP and XLP and inhibition of the fibrotic process in patients with diffuse cutaneous systemic sclerosis (dcSSc). In preclinical studies, dersimelagon exhibited the highest affinity for human MC1R compared with other melanocortin receptors, with a half maximal effective concentration (EC_50_) in the nanomolar range [5]. Dersimelagon also has been shown to induce melanogenesis in both in vitro and in vivo studies, with increased melanin production occurring in a concentration‐dependent manner in a mouse B16F1 melanoma cell line [5]. Furthermore, oral administration of dersimelagon significantly induced coat color darkening in mice and significant skin pigmentation in a reversible manner in monkeys [5].

In a recently completed phase 2 randomized, multicenter, placebo-controlled clinical trial, ENDEAVOR (ClinicalTrials.gov ID: NCT03520036), the safety and efficacy of dersimelagon at doses of 100 mg and 300 mg were investigated [6]. The results of ENDEAVOR, which included 102 patients with EPP or XLP, indicated that dersimelagon was efficacious at increasing symptom-free light exposure and had an acceptable safety and tolerability profile after 16 weeks of treatment [6]. Here, we report the results of a first-in-human phase 1 double-blind, placebo-controlled study (ClinicalTrials.gov ID: NCT02834442) that evaluated the safety, tolerability, pharmacokinetics (PK), and pharmacodynamics (PD) of single and multiple ascending oral doses of dersimelagon in healthy participants, including effects of sex, age, and race on the PK profile of dersimelagon. To complement these results, and because the information on distribution volume, clearance, and absolute bioavailability in animals is helpful in understanding the PK characteristics of dersimelagon, we also report findings from the preceding nonclinical animal studies to demonstrate the similarity between the preclinical PK profile in animals and the clinical PK profile of dersimelagon in humans. Moreover, allometric projections from animal PK data are described.

## Methods

### Study design

This trial was a phase 1 randomized, double-blind, placebo-controlled study conducted at a single center in the UK. Prior to study initiation, the protocol and all other appropriate documents were reviewed and approved by an Independent Ethics Committee and local regulatory authorities. The study was conducted in accordance with the ethical principles stated in the Declaration of Helsinki, the International Conference on Harmonisation for Good Clinical Practice guidance, and all local and regional applicable laws and regulations. Prior to the performance of any study-related assessments and procedures, all participants signed a written informed consent form with details of the trial treatment, procedures, and potential risks.

### Study participants

The study consisted of eight parts, of which only five are presented in this report (A, D, E, F, and H) and summarized in eTable 1. Eligible participants were healthy individuals aged 18 to 55 years (inclusive at screening, except for Part H) with a body weight ≥ 50 kg for females and ≥ 60 kg for males and a body mass index (BMI) ranging from 18 to 30 kg/m^2^ at the time of screening and day 1. Part A included White males, whereas Part D included White females who were not pregnant or lactating. Part F included Black males, and Part H included White males aged ≥ 65 years. Participants who had previously received dersimelagon, who had used afamelanotide or melanotan within 6 months prior to the study, or with a history of melanoma and/or dysplastic naevus were not included in the study. Randomization was performed according to a computer-generated randomization list using SAS^®^ version 9.3 (Cary, North Carolina, US) prior to the first administration of study drug.

### Study treatments

Dersimelagon was supplied as a 50-mg tablet or as a powder (1 to 600 mg, for oral suspension) of active substance, and placebo was supplied as either a tablet or powder to match dersimelagon dosage strength.

#### Single doses

Part A of the study was a single ascending dose (SAD) study conducted in seven dosed cohorts. Each cohort was composed of eight participants, and in each cohort, participants were randomized to receive dersimelagon suspension (n = 6) or placebo suspension (n = 2). In total, 56 participants were included and received dersimelagon at doses of 1 mg, 3 mg, 10 mg, 30 mg, 100 mg, 300 mg, and 600 mg. In parts D, F, and H of the study, participants received a single oral dose of either 100 mg of dersimelagon (n = 6) or placebo (n = 2) as a tablet formulation. To evaluate the effect of sex difference Part D included eight female participants, and Part F evaluated the effect of race, and included eight Black participants. Part H evaluated the effect of age in eight participants aged ≥ 65 years.

#### Multiple ascending doses

The multiple ascending dose (MAD) study (Part E) was conducted in four cohorts in which participants received multiple dose administration of dersimelagon as a tablet or suspension formulation for 14 days in one treatment period. Each cohort was composed of 12 participants who were randomized to dersimelagon (n = 9) or placebo (n = 3). The dose levels of dersimelagon used were 30 mg (Cohort 1, suspension), 150 mg (Cohort 2, tablet), 300 mg (Cohort 3, tablet), and 450 mg (Cohort 4, tablet). A Fitzpatrick scale test was performed at screening to categorize patient skin types into type I (always burns, never tans), type II (usually burns, tans with difficulty), type III (sometimes mild burn, gradually tans), type IV (rarely burns, tans with ease), type V (very rarely burns, tans very easily), or type VI (never burns, always tans) [7]. Participants with Fitzpatrick skin types II to IV were included in the 30-, 150-, and 300-mg cohorts. The protocol was amended following emerging pigmentation data, and only participants with Fitzpatrick skin type V were included in the 450-mg cohort.

### Safety assessments

Safety and tolerability were assessed by evaluating vital signs (supine blood pressure, pulse rate, respiratory rate, and body temperature), 12-lead electrocardiogram (ECG) parameters, clinical laboratory assessments, and adverse events (AEs). AEs were considered treatment emergent if they occurred after the first administration of dersimelagon or if a predose event increased in severity following dosing. The frequency and incidence of treatment-emergent AEs (TEAEs) were summarized by system organ class and preferred term for each treatment group. AEs were coded using the Medical Dictionary for Regulatory Activities (MedDRA) version 19.0. Safety laboratory test results, vital signs, and ECG parameters were summarized by treatment group and planned sampling point. All participants in the study (N = 144) were included in the safety analysis population.

### Pharmacokinetic assessments 

Blood samples for single-dose PK evaluations (Parts A, D, F, and H) were collected at predose and at 0.25, 0.5, 1, 2, 3, 4, 5, 6, 8, 10, 12, 24, and 48 h postdose and at day 5 follow-up (96 h). Blood samples for MAD evaluations (Part E) were collected on day 1 (predose and at 0.25, 0.5, 1, 2, 3, 4, 5, 6, 8, 10, and 12 h postdose), day 2 to day 13 (predose), day 14 (predose and at 0.25, 0.5, 1, 2, 3, 4, 5, 6, 8, 10, and 12 h postdose), day 15 (24 h post last dose), day 16 (48 h post last dose), and day 17 (72 h post last dose). To determine the concentrations of dersimelagon and dersimelagon glucuronide (a primary in vitro human metabolite), plasma samples were analyzed using a validated liquid chromatography coupled with tandem mass spectrometry (LC/MS/MS) method with solid phase extraction with a lower limit of quantification (LLOQ) of 0.1 ng/mL. The plasma PK parameters, including maximum observed plasma concentration (C_max_), time to maximum plasma concentration (T_max_), plasma terminal elimination half-life (t_½_), area under the plasma concentration–time curve from time zero to the last measurable concentration (AUC_0-last_), to infinity (AUC_0-∞_), and over the 24-h dosing interval, at steady state (AUC_0-τ_) were calculated with reference to day 1 dosing and/or the last day of dosing where applicable for dersimelagon and its metabolite dersimelagon glucuronide. The PK parameters were derived by noncompartmental analysis using Phoenix WinNonlin® software version 6.3 (Certara, LP, Princeton, New Jersey, US). For the calculation of PK parameters, data below the LLOQ were imputed a value of zero. Participants who received at least one dose of dersimelagon and who had at least one postdose value of plasma concentration (N = 110) were included in the PK analysis population.

### Pharmacodynamic assessments

The effect of dersimelagon treatment on pigmentation was assessed in all participants in Part A by measuring melanin density on three skin segments (lower back, forehead, and cheek) by spectrophotometry (Konica Minolta Spectrophotometry) prior to dersimelagon administration and at days 3, 5, and 15 following a single oral dose of dersimelagon. In Part E, melanin density was measured prior to dersimelagon administration and at days 3, 5, 7, 10, 15, 22, 29, and 57 by spectrophotometry on the lower back, forehead, cheek, neck, inner upper arm, and outer forearm. The melanin density was calculated according to the following formula:

$$\begin{aligned}\mathrm{Melanin \;density \,(MD_{400})} = &\;100\, [0.035307\\& + 0.009974\, \mathrm{(R_{420}- R_{400})}]\end{aligned}$$

where R_400_ is the reflection at 400 nm and R_420_ is the reflection at 420 nm [8, 9]. Participants who received at least one dose of dersimelagon or placebo and who had at least one postdose value of melanin density (Part A: N = 56; Part E: N = 48) were included in the PD analysis population.

### Statistical methods

The statistical analysis was performed using SAS^®^ version 9.4 (SAS Institute Inc.; Cary, North Carolina, US). All variables were summarized by dose level for the SAD and MAD parts and by sex, age, and race category as appropriate. Unless otherwise stated, continuous data were summarized descriptively including N (number of participants), n (number of observations), mean, standard deviation, minimum, median, and maximum. Categorical data were summarized using frequency tables (frequency and percent). A linear model was used to analyze log-transformed AUC (AUC_0-∞_) and C_max_, with race (White vs Black), sex (male vs female), or age (≤ 55 years vs ≥ 65 years) as fixed effects. The difference in least squares (LS) means and corresponding 90% CIs were back-transformed to obtain the estimates and CIs of the geometric mean ratios comparing Black with White, female with male, and participants aged ≥ 65 years with those who were aged ≤ 55 years. In Part A (SAD) and Part E (MAD), PK parameters of interest were used to evaluate dose proportionality. A linear model was used to fit the power model after log transformation of the parameter of interest. The model included the log-transformed dose as a fixed effect. The point estimate and its 95% CI were derived for slope β to evaluate dose proportionality. The results were considered dose proportional if the CI included 1.

### Nonclinical animal studies

All animal studies were performed according to the rules for the proper conduct of animal experiments and approved by the Institutional Animal Care and Use Committee of the test facility. Additional details regarding the nonclinical study protocols can be found in the Supplementary methods.

#### PK study in rats

A study was conducted to characterize the PK of dersimelagon in male Sprague–Dawley rats (n = 4) after a single oral administration (0.3, 1, and 3 mg/kg) and intravenous administration (2 mg/kg).

#### PK study in monkeys

A study was conducted to evaluate the PK of dersimelagon up to 48 h when administered orally (3 mg/kg) and intravenously (1 mg/kg) to male cynomolgus monkeys (n = 4).

#### In vitro plasma protein binding of dersimelagon

In vitro binding of radiolabeled [^14^C] dersimelagon free base to plasma proteins of rats, cynomolgus monkeys, and humans was examined by the equilibrium dialysis method. The [^14^C] dersimelagon free base (0.1, 1, and 9 µg/mL)-spiked plasma was incubated in a rapid equilibrium dialysis device for 8 h at 37 °C in a CO_2_ incubator.

#### Allometric scaling

The prediction of human clearance (CL_h_) from animal PKs was performed with several allometric approaches, including simple allometry, allometry with bile flow correction factor [10], allometry with the rule of exponent [11], allometry with unbound clearance approach [12], and fu-corrected intercept method [13]. The human distribution volume was predicted by simple allometry. The human bioavailability (F) was calculated employing the following equation:

1 $$\mathrm{F = F_{a} \times F_{g} \times F_{h}}$$

where F_a_, F_g_, and F_h_ are fractions of orally administered drugs absorbed from the intestine, intestinal availability, and hepatic availability, respectively. Human F_a_ was assumed to be equivalent to rat F_a_ (0.64, which was calculated from a mass balance study in rats). Human F_g_ was assumed to be 1.0, because dersimelagon was very stable in human intestinal microsomes. Human F_h_ was calculated from the following equation:

2 $$\mathrm{F_{h} = 1 - CL_{h} \,/\, R_{b} \,/ \,Q_{h}}$$

where R_b_ (blood/plasma concentration ratio) was 0.6 (obtained by in vitro studies), and Q_h_ (hepatic blood flow rate) was 1200 mL/h/kg. Based on these parameters, the plasma concentration–time profile at an oral dose of 100 mg in humans was simulated using 2-compartment model with 70 kg as the body weight and 1.0 h^−1^ as absorption rate constant, and the resultant C_max_ and AUC values were obtained.

## Results

### Study participants

A total of 144 participants were randomized, of whom 143 completed the study. A total of 110 participants were treated with at least one dose of dersimelagon, and 34 participants were treated with a placebo. In parts A, D, F, and H, participants (*n* = 54) were exposed to single doses of dersimelagon ranging from 1 to 600 mg. In the MAD study (Part E), participants (*n* = 36) were treated with multiple doses of dersimelagon ranging from 30 to 450 mg. The baseline demographics of study participants are summarized in Table 1. All participants were male, except for eight female participants in Part D. Mean age, weight, and BMI were generally similar across treatment groups within each part of the study, with the exception of female participants, who had a slightly lower body weight than other participants. The baseline Fitzpatrick skin type of participants in the MAD study (Part E) is summarized in eTable 2. Of note, 88.9% of participants who received 450 mg of dersimelagon in the MAD study identified as Black, and all were Fitzpatrick skin type V, based on emerging pigmentation data obtained from previous cohorts in Part E.

**Table 1:** Baseline demographics of study participants investigated for single ascending dose (Part A), effect of sex (Part D), effect of age (Part H), effect of race (Part F), and multiple ascending doses (Part E)

	**Treatment**	***N***	**Sex**		**Age (years)** **mean (SD)**	**Ethnicity**		**Race**			**Weight (kg)** **mean (SD)**	**BMI (kg/m**^**2**^**)** **mean (SD)**
			**Male** ***n* (%)**	**Female** ***n* (%)**		**Hispanic/Latino *n* (%)**	**Not Hispanic/Latino** ***n* (%)**	**White** ***n* (%)**	**Black** ***n* (%)**	**Other** ***n* (%)**		
**Single ascending dose** **(Part A)**	**Placebo**	**14**	14 (100.0)	0 (0)	37.1 (8.86)	0 (0)	14 (100.0)	14 (100.0)	0 (0)	0 (0)	77.04 (9.45)	24.38 (2.74)
	**Dersimelagon 1 mg**	**6**	6 (100.0)	0 (0)	35.2 (8.64)	0 (0)	6 (100.0)	6 (100.0)	0 (0)	0 (0)	78.62 (11.48)	24.95 (2.74)
	**Dersimelagon 3 mg**	**6**	6 (100.0)	0 (0)	29.3 (5.92)	0 (0)	6 (100.0)	6 (100.0)	0 (0)	0 (0)	85.48 (12.58)	26.13 (2.27)
	**Dersimelagon 10 mg**	**6**	6 (100.0)	0 (0)	33.3 (11.64)	0 (0)	6 (100.0)	6 (100.0)	0 (0)	0 (0)	84.97 (15.81)	25.83 (3.23)
	**Dersimelagon 30 mg**	**6**	6 (100.0)	0 (0)	27.5 (4.76)	0 (0)	6 (100.0)	6 (100.0)	0 (0)	0 (0)	74.55 (6.26)	23.58 (2.27)
	**Dersimelagon 100 mg**	**6**	6 (100.0)	0 (0)	36.3 (11.02)	0 (0)	6 (100.0)	6 (100.0)	0 (0)	0 (0)	72.80 (13.64)	22.42 (3.29)
	**Dersimelagon 300 mg**	**6**	6 (100.0)	0 (0)	33.3 (7.37)	0 (0)	6 (100.0)	6 (100.0)	0 (0)	0 (0)	74.48 (5.58)	24.78 (2.74)
	**Dersimelagon 600 mg**	**6**	6 (100.0)	0 (0)	43.0 (10.30)	0 (0)	6 (100.0)	6 (100.0)	0 (0)	0 (0)	74.27 (11.11)	24.03 (4.06)
**Effect of sex** **(Part D)**	**Placebo**	**2**	0 (0)	2 (100.0)	40.0 (21.21)	0 (0)	2 (100.0)	2 (100.0)	0 (0)	0 (0)	65.30 (3.68)	24.40 (0.14)
	**Dersimelagon 100 mg**	**6**	0 (0)	6 (100.0)	35.5 (9.14)	0 (0)	6 (100.0)	6 (100.0)	0 (0)	0 (0)	64.27 (11.59)	23.83 (4.37)
**Effect of age** **(Part H)**	**Placebo**	**2**	2 (100.0)	0 (0)	67.5 (3.54)	0 (0)	2 (100.0)	2 (100.0)	0 (0)	0 (0)	82.30 (3.54)	29.50 (0.28)
	**Dersimelagon 100 mg**	**6**	6 (100.0)	0 (0)	68.0 (4.47)	0 (0)	6 (100.0)	6 (100.0)	0 (0)	0 (0)	76.65 (10.21)	25.33 (2.73)
**Effect of race** **(Part F)**	**Placebo**	**2**	2 (100.0)	0 (0)	36.5 (10.61)	0 (0)	2 (100.0)	0 (0)	2 (100.0)	0 (0)	95.35 (7.845)	26.85 (1.623)
	**Dersimelagon 100 mg**	**6**	6 (100.0)	0 (0)	31.2 (8.42)	0 (0)	6 (100.0)	0 (0)	6 (100.0)	0 (0)	73.75 (12.42)	23.53 (2.25)
**Multiple ascending dose (Part E)**	**Placebo**	**12**	12 (100.0)	0 (0)	35.8 (7.98)	2 (16.7)	10 (83.3)	9 (75.0)	2 (16.7)	1 (8.3)	78.66 (10.57)	25.21 (2.79)
	**Dersimelagon 30 mg**	**9**	9 (100.0)	0 (0)	37.8 (8.70)	1 (11.1)	8 (88.9)	8 (88.9)	0 (0)	1 (11.1)	75.60 (8.49)	24.68 (2.71)
	**Dersimelagon 150 mg**	**9**	9 (100.0)	0 (0)	36.7 (9.27)	0 (0)	9 (100.0)	9 (100.0)	0 (0)	0 (0)	80.10 (9.31)	25.13 (3.02)
	**Dersimelagon 300 mg**	**9**	9 (100.0)	0 (0)	35.8 (9.98)	0 (0)	9 (100.0)	9 (100.0)	0 (0)	0 (0)	77.58 (10.83)	24.42 (2.22)
	**Dersimelagon 450 mg**	**9**	9 (100.0)	0 (0)	34.8 (9.01)	1 (11.1)	8 (88.9)	0 (0)	8 (88.9)	1 (11.1)	80.87 (8.05)	25.27 (2.13)

Percentages are based on the number of participants in each treatment group

*BMI* body mass index, *n* number of observations, *N* number of participants

### Safety and tolerability

The TEAEs reported by at least two participants in any treatment group are summarized in Table 2. Overall, the incidence of TEAEs was relatively low, with most events occurring during Part A (SAD) and Part E (MAD) and the majority being mild to moderate in severity. No deaths or serious AEs were reported during any part of the study. Single oral doses of dersimelagon were generally well tolerated at levels of 1 to 600 mg. In Part A (SAD), 24 TEAEs were reported by 13 (31.0%) participants receiving dersimelagon, and three TEAEs were reported by three (21.4%) participants receiving placebo. The most common TEAEs experienced in Part A were headache and nausea, each reported in two (4.8%) participants, and contact dermatitis in three (7.1%) participants. Of these TEAEs, only headache and nausea were considered possibly related to study drug. In Part D (effect of sex), there was one TEAE experienced by one participant receiving placebo and none reported by those receiving dersimelagon. No TEAEs were reported during Parts F (effect of race) or H (effect of age). In Part E (MAD), 151 TEAEs were experienced by 34 (94.4%) participants receiving dersimelagon, and 10 TEAEs were experienced by four (33.3%) participants receiving placebo.

**Table 2:** Incidence of treatment-emergent adverse events occurring in at least 2 participants in any treatment group in SAD and MAD studies

***n* (%)**	**Single ascending dose (Part A)**									**Multiple ascending dose (Part E)**					
	**Placebo** **(*N* = 14)**	**Dersimelagon**								**Placebo** **(*N* = 12)**	**Dersimelagon**				
		**1 mg** **(*N* = 6)**	**3 mg** **(*N* = 6)**	**10 mg** **(*N* = 6)**	**30 mg** **(*N* = 6)**	**100 mg** **(*N* = 6)**	**300 mg** **(*N* = 6)**	**600 mg** **(*N* = 6)**	**Total** **(*N* = 42)**		**30 mg** **(*N* = 9)**	**150 mg** **(*N* = 9)**	**300 mg** **(*N* = 9)**	**450 mg** **(*N* = 9)**	**Total** **(*N* = 36)**
**Any TEAE**	3 (21.4%)	2 (33.3%)	0 (0.0%)	2 (33.3%)	3 (50.0%)	1 (16.7%)	2 (33.3%)	3 (50.0%)	13 (31.0%)	4 (33.3%)	8 (88.9%)	8 (88.9%)	9 (100.0%)	9 (100.0%)	34 (94.4%)
**Nasopharyngitis**	-	-	-	-	-	-	-	-	-	-	1 (11.1%)	2 (22.2%)	-	-	3 (8.3%)
**Melanocytic naevus**	-	-	-	-	-	-	-	-	-	-	-	4 (44.4%)	6 (66.7%)	-	10 (27.8%)
**Dizziness**	-	-	-	-	-	-	-	-	-	-	1 (11.1%)	-	-	1 (11.1%)	2 (5.6%)
**Headache**	-	-	-	-	1 (16.7%)	-	-	1 (16.7%)	2 (4.8%)	1 (8.3%)	3 (33.3%)	-	2 (22.2%)	4 (44.4%)	9 (25.0%)
**Nausea**	-	-	-	-	-	-	-	2 (33.3%)	2 (4.8%)	-	-	-	-	-	-
**Constipation**	-	-	-	-	-	-	-	-	-	2 (16.7%)	-	-	1 (11.1%)	-	1 (2.8%)
**Tongue pigmentation**	-	-	-	-	-	-	-	-	-	-	-	-	-	2 (22.2%)	2 (5.6%)
**Dermatitis contact**	-	1 (16.7%)	-	-	1 (16.7%)	-	-	1 (16.7%)	3 (7.1%)	-	-	-	1 (11.1%)	1 (11.1%)	2 (5.6%)
**Dermatitis acneiform**	-	-	-	-	-	-	-	-	-	-	-	-	-	3 (33.3%)	3 (8.3%)
**Dry skin**	-	-	-	-	-	-	-	-	-	-	-	-	-	3 (33.3%)	3 (8.3%)
**Ephelides**	-	-	-	-	-	-	-	-	-	-	-	-	6 (66.7%)	-	6 (16.7%)
**Hair color changes**	-	-	-	-	-	-	-	-	-	-	-	-	2 (22.2%)	-	2 (5.6%)
**Lentigo**	-	-	-	-	-	-	-	-	-	1 (8.3%)	6 (66.7%)	7 (77.8%)	6 (66.7%)	-	19 (52.8%)
**Skin hyperpigmentation**	-	-	-	-	-	-	-	-	-	1 (8.3%)	2 (22.2%)	2 (22.2%)	7 (77.8%)	7 (77.8%)	18 (50.0%)
**Arthralgia**	-	-	-	-	-	-	-	-	-	-	1 (11.1%)	-	-	2 (22.2%)	3 (8.3%)
**Back pain**	-	-	-	-	-	-	-	-	-	-	-	1 (11.1%)	-	2 (22.2%)	3 (8.3%)
**Vessel puncture site bruise**	-	-	-	-	-	-	-	-	-	1 (8.3%)	-	1 (11.1%)	1 (11.1%)	-	2 (5.6%)
**Procedural pain**	-	-	-	-	-	-	-	-	-	-	-	-	1 (11.1%)	1 (11.1%)	2 (5.6%)

TEAEs are defined as events that started on or after the date of first dose of study drug. Total column includes all active dose levels. A dash (-) indicates there were no TEAEs reported

*n* number of participants meeting specified criteria, *N* number of participants in treatment group, *TEAE* treatment-emergent adverse event

Overall, participants who received multiple doses (Part E) had a higher incidence of TEAEs than those who received a single dose (Parts A, D, F, and H). The most common TEAEs experienced in participants receiving multiple doses of dersimelagon were related to skin pigmentation, including lentigo (*n* = 20), skin hyperpigmentation (*n* = 19), and melanocytic naevus (*n* = 10). Two cases of melanocytic naevus reported by participants receiving 300 mg of dersimelagon were considered severe; however, both cases were confirmed to be nonmalignant. There were no safety concerns based on laboratory parameters, vital signs, ECG, and physical examination for any group during the study.

### Pharmacokinetics of dersimelagon

#### Single ascending dose study (Part A)

**Fig. 1:**
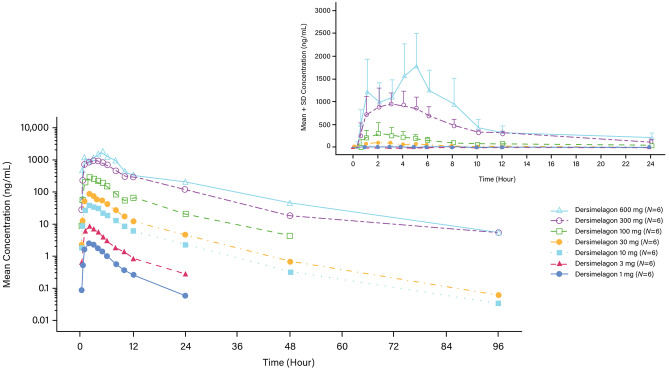
Mean plasma concentrations of dersimelagon following single ascending doses (Part A)

Following single oral doses of 1, 3, 10, 30, 100, 300, and 600 mg, dersimelagon was rapidly absorbed, with median T_max_ values generally similar at each dose, ranging from 1.99 to 5.00 h postdose (Table 3). After reaching C_max_, the plasma concentrations of dersimelagon appeared to decline in a biphasic manner (Fig. 1). The mean t_½_ of dersimelagon was generally similar across the 10- to 600-mg doses, with values ranging from 7.63 to 10.58 h. At the lower dose levels of 1 and 3 mg, the mean t_½_ was shorter, ranging between 5.14 to 6.43 h, due to the lack of point estimates in the terminal phase for the estimation of the elimination rate constant (k_el_). Dose-normalized plasma PK parameters are presented in eFig. 1, and analysis of dose proportionality is presented in eTable 3. The slope estimates (95% CI) from the regression analysis for AUC_0-∞_ and C_max_ were 1.12 (1.07–1.17) and 1.05 (1.00–1.10), respectively, for dersimelagon. The lower limit of the 95% CI above unity indicated that the systemic exposure to dersimelagon in plasma, based on AUC_0-∞_ and C_max_ values, appeared to increase in a slightly more than dose-proportional manner over the 1- to 600-mg single dose range (eTable 3).

**Table 3:** Pharmacokinetic parameters of a single oral dose of dersimelagon in healthy adult volunteers

**PK variable**	**Statistical parameter**	**Single ascending dose (Part A)**							**Effect of sex** **(Part D)**		**Effect of age** **(Part H)**		**Effect of race** **(Part F)**	
		**1 mg** **(*N* = 6/*n* = 6)**	**3 mg** **(*N* = 6/*n* = 6)**	**10 mg** **(*N* = /*n* = 66)**	**30 mg** **(*N* = 6/*n* = 6)**	**100 mg** **(*N* = 6/*n* = 6)**	**300 mg** **(*N* = 6/*n* = 6)**	**600 mg** **(*N* = 6/*n* = 6)**	**Male**^**a**^ **100 mg** **(*N* = 6/*n* = 6)**	**Female** **100 mg** **(*N* = 6/*n* = 6)**	** ≤ 55 years**^**a**^ **100 mg** **(*N* = 6/*n* = 6)**	** ≥ 65 years** **100 mg** **(*N* = 6/*n* = 6)**	**White**^**a**^ **100 mg** **(*N* = 6/*n* = 6)**	**Black** **100 mg** **(*N* = 6/*n* = 6)**
**AUC**_**0-∞**_ **(ng∙h/mL)**	**Mean (SD)**	15.5 (6.82)	52.7 (11.2)	313.1 (78.1)	664.6 (182.3)	2588.2 (1323.9)	11,553.6 (3234.4)	18,836.1 (5814.5)	2588.2 (1323.9)	4214.8 (1588.1)	2588.2 (1323.9)	3122.2 (1113.5)	2588.2 (1323.9)	2633.6 (1293.0)
**C**_**max**_** (ng/mL)**	**Mean (SD)**	2.50 (0.83)	8.76 (1.86)	38.1 (7.56)	86.5 (23.6)	339.6 (230.8)	1183.4 (316.0)	2103.8 (401.2)	339.6 (230.8)	448.7 (220.5)	339.6 (230.8)	407.1 (114.1)	339.6 (230.8)	316.7 (153.9)
**T**_**max**_** (h)**	**Median** **(range)**	2.50 (1.98, 3.03)	2.00 (1.00, 2.00)	2.00 (1.98, 4.00)	2.00 (1.98, 5.00)	3.00 (1.00, 6.02)	1.99 (0.98, 4.98)	5.00 (0.98, 5.00)	3.00 (1.00, 6.02)	5.00 (1.00, 6.00)	3.00 (1.00, 6.02)	2.60 (0.98, 4.98)	3.00 (1.00, 6.02)	3.99 (1.97, 5.00)
**t**_**1/2**_** (h)**	**Mean (SD)**	5.14 (3.43)	6.43 (2.17)	8.27 (2.69)	7.63 (1.49)	9.77 (2.26)	9.49 (1.72)	10.6 (3.95)	9.77 (2.26)	12.1 (5.60)	9.77 (2.26)	12.1 (2.80)	9.77 (2.26)	9.25 (3.97)

*AUC*_*0-∞*_ area under the plasma concentration versus time curve from time zero to infinity, *C*_*max*_ maximum observed plasma concentration, *N* number of participants, *n* number of observations, *t*_*1/2*_ terminal elimination half-life, *T*_*max*_ time to maximum plasma concentration

^a^Comparator group is composed of participants from Part A

Dersimelagon was rapidly metabolized to dersimelagon glucuronide, a major metabolite of dersimelagon, and demonstrated a similar PK profile to dersimelagon, with a median T_max_ similar to dersimelagon across dose levels (between 1 and 5 h postdose); however, the systemic exposures to dersimelagon glucuronide based on C_max_ and AUC_0-∞_ were extremely low compared with that of unchanged dersimelagon (ratio of C_max_ or AUC_0-∞_ of dersimelagon glucuronide to that of unchanged dersimelagon: ≤ 0.05; data not shown).

#### Effect of sex (Part D), age (Part H), and race (Part F)

Following a single oral dose, 100 mg of dersimelagon was rapidly absorbed in all demographic subgroups, with T_max_ occurring at similar times across comparator subgroups (T_max_ = 3 vs 5 h in males vs females; 3 vs 2.6 h in participants ≤ 55 years vs participants ≥ 65 years; 3 vs 3.99 h in White vs Black participants; Table 3; eFig. 2). The mean t_½_ values for dersimelagon were also similar across comparator subgroups, indicating comparable elimination profiles regardless of demographics.

Both C_max_ and AUC_0-∞_ were higher in female participants compared with those in males; however, only the difference in AUC_0-∞_ was significant based on the 90% CI of the ratio of female over male since 90% CIs did not include the value 1 (Table 4). In participants ≥ 65 years, geometric LS mean C_max_ and AUC_0-∞_ were approximately 37% and 25% higher, respectively, compared with participants ≤ 55 years, but these differences were not statistically significant. Geometric LS mean C_max_ and AUC_0-∞_ were similar in Black and White participants, and differences were not statistically significant.

**Table 4:** ANOVA analysis between PK parameters and effect of sex, age, and race on exposure

**Part D (sex)**	**Parameter**	**LS mean (male)** ***N* = 6/*n* = 6**	**LS mean (female)** ***N* = 6/*n* = 6**	**Ratio of female over male**	**90% CI lower**	**90% CI upper**
	C_max_ (ng/mL)	287.3	405.2	1.41	0.79	2.53
	AUC_0-∞_ (ng·h/mL)	2353.1	3983.5	1.69	1.09	2.62
**Part H (age)**	**Parameter**	**LS mean (≤ 55 years)** ***N* = 6/*n* = 6**	**LS mean (≥ 65 years)** ***N* = 6/*n* = 6**	**Ratio of ≥ 65 years over ≤ 55 years**		
	C_max_ (ng/mL)	287.3	393.8	1.37	0.83	2.26
	AUC_0-∞_ (ng·h/mL)	2353.1	2947.6	1.25	0.80	1.96
**Part F (race)**	**Parameter**	**LS mean (White)** ***N* = 6/*n* = 6**	**LS mean (Black)** ***N* = 6/*n* = 6**	**Ratio of Black over White**		
	C_max_ (ng/mL)	287.3	281.6	0.98	0.53	1.81
	AUC_0-∞_ (ng·h/mL)	2353.1	2374.9	1.01	0.61	1.68

*ANOVA* analysis of variance, *AUC*_*0-∞*_ area under the plasma concentration–time curve from time zero to infinity, *C*_*max*_ maximum observed plasma concentration, *LS* least squares, *N* number of participants, *n* number of observations, *PK* pharmacokinetic

#### Multiple ascending dose study (Part E)

Following multiple doses at 30, 150, 300, and 450 mg, dersimelagon was rapidly absorbed, with median T_max_ values ranging from 4 to 5 h postdose on days 1 and 14. The mean t_1/2_ values ranged from 6.28 to 15.55 h on day 1 and 10.56 to 18.97 h on day 14 (Table 5). The day 14 slope estimates (95% CI) from the regression analysis for AUC_0-τ_ and C_max_ were 1.26 (1.14–1.39) and 1.25 (1.13–1.36), respectively, for dersimelagon. The lower limit of the 95% CIs of the slopes were over unity, indicating a slightly more than dose-proportional increase in the systemic exposure over the dose range of 30 to 450 mg (eTable 3; eFig. 3). There was a slight accumulation of dersimelagon following multiple dosing on day 14 compared to day 1, with mean ratio of accumulation (RA) values ranging from 1.14 to 1.82 across the 30- to 450-mg dose range while the mean linearity factor (LF) values were 1.07 to 1.53 over this dose range. The mean trough concentration ratios were over 0.95 after 5 days of multiple dosing at each dose level, suggesting that steady state was generally reached by day 5 of multiple dosing (eFig. 4). The PK profile of dersimelagon glucuronide followed a similar trend, with a median T_max_ and mean t_1/2_ similar to dersimelagon across dose levels on days 1 and 14. However, as shown in Part A (SAD study), the systemic exposure to dersimelagon glucuronide was extremely low compared with that of unchanged dersimelagon (data not shown).

**Table 5:** Pharmacokinetic parameters of multiple doses of dersimelagon in healthy adult volunteers

**PK variable**	**Statistical parameter**	**Visit**	**Multiple ascending dose (Part E)**			
			**30 mg** **(*n* = 9)**	**150 mg** **(*n* = 9)**	**300 mg** **(*n* = 9)**	**450 mg** **(*n* = 9)**
**C**_**max**_** (ng/mL)**	**Mean (SD)**	**Day 1**	79.31 (22.2)	507.53 (221.9)	1139.36 (331.3)	1855.39 (1337.6)
		**Day 14**	85.40 (26.0)	537.79 (202.1)	1389.00 (315.3)^b^	2398.50 (546.6)^c^
**T**_**max**_** (h)**	**Median (range)**	**Day 1**	4.00 (2.97, 5.02)	4.98 (2.03, 6.00)	4.93 (1.98, 5.97)	5.00 (4.97, 6.02)
		**Day 14**	4.00 (3.00, 5.00)	5.00 (3.02, 6.02)	4.02 (3.00, 4.97)^b^	4.49 (2.00, 5.02)^c^
**t**_**1/2**_** (h)**	**Mean (SD)**	**Day 1**	6.28 (2.0)	8.48 (3.5)	9.60 (3.2)	15.55 (12.0)
		**Day 14**	10.81 (2.9)	10.56 (2.8)	14.35 (6.2)^b^	18.97 (10.1)^c^
**AUC (ng·h/mL)**^**a**^	**Mean (SD)**	**Day 1**	638.48 (199.50)	3790.10 (1466.4)	8596.39 (1982.5)	16,854.30 (6815.1)
		**Day 14**	680.31 (218.1)	4379.56 (1385.9)	11,926.64 (2740.3)^b^	19,256.99 (9218.2)^c^
**Linearity factor**	**Mean (SD)**	**AUC**_**0-24**_** day 14 / AUC**_**0-∞**_** day 1**	1.07 (0.15)	1.19 (0.21)	1.53 (0.45)^b^	1.36 (0.67)^c^
**Ratio of accumulation**	**Mean (SD)**	**AUC**_**0-24**_** day 14 / AUC**_**0-24**_** day 1**	1.14 (0.14)	1.36 (0.18)	1.71(0.40)^b^	1.82 (0.64)^c^

*AUC*_*0-∞*_ area under the plasma concentration–time curve from time zero to infinity, *AUC*_*0-τ*_ area under the plasma concentration–time curve over the dosing interval, *C*_*max*_ maximum observed plasma concentration, *n* number of observations, *t*_*½*_ apparent plasma terminal elimination half-life, *T*_*max*_ time to maximum plasma concentration

^a^AUC for day 1: AUC_0-∞_, AUC for day 14: AUC_0-τ_

^b^Calculated on the basis of *n* = 3 observations

^c^Calculated on the basis of *n* = 8 observations

### Pharmacodynamics of dersimelagon

**Fig. 2:**
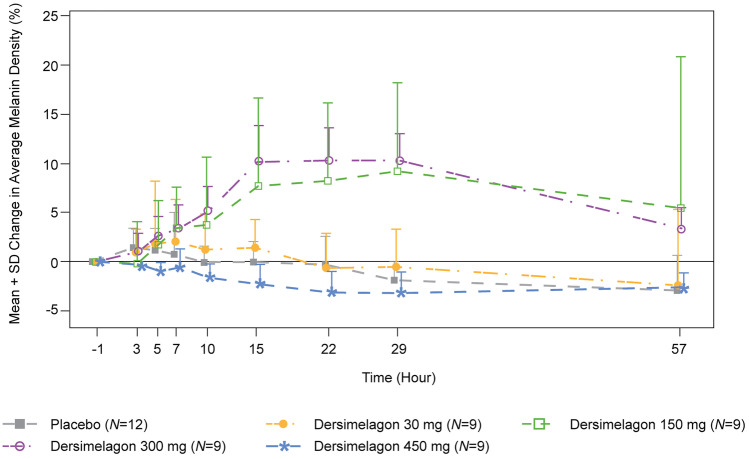
Mean percent change from baseline in average melanin density over time (Part E). Note: In participants receiving 300 mg of dersimelagon, 4 did not take study drug on day 14, and 2 did not take study drug on days 13 and 14. In participants receiving 450 mg of dersimelagon, all were Fitzpatrick skin type V, and 1 participant did not take study drug on days 12, 13, and 14

No treatment-related effects on melanin density were observed following single doses of dersimelagon (Part A). In Part E, apparent treatment-related increases in melanin density during the treatment period were observed in participants receiving 150 and 300 mg of dersimelagon compared with those receiving placebo (Fig. 2). At day 15, mean changes from baseline in the average melanin density were 0.07%, 1.43%, 7.71%, 10.22%, and –2.11% in the placebo, 30-mg, 150-mg, 300-mg, and 450-mg dersimelagon groups, respectively, and did not reach a plateau during this time frame. The increases in melanin density were maintained in the 150- and 300-mg dersimelagon groups up to day 29, with signs of reversibility observed at day 57.

### Pharmacokinetics in rats and monkeys

Dersimelagon was rapidly absorbed in rats and monkeys following oral administration. The bioavailability was 7.2% and 6.9% at 1 and 3 mg/kg in rats, respectively, and 31% in monkeys (eTable 4). The t_1/2_ was approximately 2 h in rats and 5 h in monkeys. Of note, C_max_ and AUC_0–last_ increased in a greater than dose-proportional manner between 0.3 and 1 mg/kg and tended to increase with doses between 1 and 3 mg/kg when assessed in rats.

### Plasma protein binding of dersimelagon

The in vitro plasma protein binding of dersimelagon free base ([^14^C] dersimelagon free base) in rats, cynomolgus monkeys, and humans is shown in eTable 5. The plasma protein bindings of [^14^C] dersimelagon free base at concentrations of 0.1, 1, and 9 µg/mL were 98.3%, 98.2%, and 97.8% in rats; 97.2%, 96.7%, and 97.3% in cynomolgus monkeys; and 98.3%, 98.4%, and 98.2% in humans, respectively.

### Interspecies scaling

The human C_max_ and AUC at an oral dose of 100 mg of dersimelagon predicted from animal PK data are shown in Table 6 in comparison to actual results in humans. The predicted C_max_ by several allometric methods was within threefold of actual C_max_, and the predicted AUC was generally estimated to be lower than the actual AUC, except for allometry with unbound clearance approach, which was almost equal to actual AUC.

**Table 6:** Predicted and actual mean values of C_max_ and AUC of dersimelagon after 100 mg oral administration in humans

**Model**	**C**_**max**_ **(ng/mL)**	**Ratio of C**_**max**_ **(predicted/actual)**	**AUC (ng/mL)**	**Ratio of AUC** **(predicted/actual)**
**Actual**	339.6	-	2588.2	-
**Simple allometry**	203.9	0.60	463.8	0.18
**Allometry with bile flow correction factor**	363.1	1.07	901.4	0.35
**Allometry with the rule of exponent**	250.3	0.74	583.9	0.23
**Allometry with unbound clearance approach**	957.3	2.82	3400.9	1.31
**Fu-corrected intercept method**	595.6	1.75	1689.0	0.65

*AUC* area under the plasma concentration–time curve, *C*_*max*_ maximum observed plasma concentration; *fu*, fraction unbound

## Discussion

The present report describes the first-in-human SAD and MAD trial of oral dersimelagon conducted in healthy volunteers. Dersimelagon was generally well tolerated following single oral doses between 1 and 600 mg and multiple oral doses between 30 and 450 mg, including in female, Black, and older participants. The most commonly reported TEAEs in participants receiving multiple doses of dersimelagon (Part E) were related to skin pigmentation changes, including lentigo and skin hyperpigmentation. These TEAEs are similar to those previously reported in the phase 2 ENDEAVOR study [6], which investigated the safety and efficacy of dersimelagon in patients with EPP and XLP, with the most common TEAEs being nausea (27.9%), ephelides (23.5%), and skin hyperpigmentation (20.6%). These TEAEs related to changes in skin pigmentation may be attributable to the study drug based on the pharmacological effects of dersimelagon by virtue of its mechanism of action as a selective MC1R agonist.

The pharmacokinetics of dersimelagon in rats and monkeys following intravenous administration indicated a moderate volume of distribution, clearance, and half-life. Oral bioavailability was low in rats and moderate in monkeys. In the clinical trial, dersimelagon was rapidly absorbed following single oral doses, with exposure appearing to increase in a slightly more than dose-proportional manner, similar to that observed in rats following oral administration of dersimelagon. Further, similar PK properties of rapid absorption and high plasma protein binding were observed for dersimelagon in both humans and animals from the present study. In the drug discovery process, the prediction of human PK of a new chemical entity is routinely performed using animal PK data. In this report, the extent to which the predicted human PK calculated from animal PK data of dersimelagon was consistent with the actual human PK was retrospectively investigated. The predicted human C_max_ values were generally in good agreement with the actual human C_max_ and were within twofold of the actual C_max_ except for the unbound clearance approach method. The predicted human AUC values ranged from one-third to one-fifth of actual AUC for 3 methods, but were within twofold of actual AUC for the other two methods. Overall, the usefulness of commonly used human PK prediction methods was demonstrated.

There were no statistically significant effects of age or race on the PK profile of dersimelagon; however, increased exposure was observed in females (vs males). This difference can most likely be attributed to differences in the body weight generally observed in male versus female participants. Our findings suggest that future development of a population PK model will be useful to evaluate the influence of factors such as body weight, sex, race, and ethnicity on the PK profile of dersimelagon. Furthermore, based on the mean trough concentration ratios, steady state was generally reached by 5 days of multiple dosing, which may be indicative of the time frame necessary to see any treatment effects of dersimelagon.

No treatment-related effects on melanin density were observed following single doses of dersimelagon (Part A). However, in the MAD study (Part E), treatment-related increases in melanin density were observed in the 150- and 300-mg groups compared with the placebo group during the treatment period. This is consistent with previous preclinical reports in which selective activation of MC1R by dersimelagon led to increases in skin pigmentation (eumelanin production) in both in vitro and in vivo animal studies [5]. To our knowledge, dersimelagon is the first orally available compound that has demonstrated increases in melanin density in humans. Of note, increases in melanin density were not observed in the 450-mg group. Only participants with Fitzpatrick skin type V were included in the 450-mg cohort, limiting comparison of PD effects of dersimelagon in this cohort. Taken together, the findings of this study suggest that dersimelagon may have the potential to increase pain-free light exposure in patients with EPP and XLP due to its potential photoprotective effects.

In a recently completed phase 2, randomized, multicenter, placebo-controlled clinical trial (ENDEAVOR), oral administration of dersimelagon to patients with EPP or XLP was shown to increase symptom-free light exposure with an acceptable safety and tolerability profile after 16 weeks of treatment [6]. Currently, the only approved pharmacological intervention indicated for use in patients with EPP is afamelanotide (an MC1R agonist) administered as a subcutaneous implant [4]. The availability of dersimelagon as an oral formulation will confer advantages, namely noninvasiveness and convenience, for the treatment of patients with EPP or XLP.

## Conclusions

In this first-in-human phase 1 study, dersimelagon was generally well tolerated following oral administration and demonstrated a generally consistent PK profile across diverse groups of healthy participants. An effective and safe orally administered treatment will address an important unmet need for patients with EPP and XLP, especially for pediatric and adolescent patients who have had no approved pharmacological options to date. Treatment-related increases in melanin density following multiple doses of dersimelagon support further investigation into the photoprotective effects of dersimelagon. A recently completed phase 3 study (ClinicalTrials.gov ID: NCT04402489) evaluated the efficacy and safety of dersimelagon in adults and adolescents with EPP and XLP.

## Supplementary Information

Below is the link to the electronic supplementary material.

Supplementary file1 (PDF 550 KB)

## Data Availability

The datasets generated during and/or analyzed in this study are available from the corresponding author upon reasonable request.
