# Supplementary material for: Results from a first-in-human study of dersimelagon, an investigational oral selective MC1R agonist
Source: Eur J Clin Pharmacol. 2023 Apr 15;79(6):801–13. doi: 10.1007/s00228-023-03476-6 (PMC10229459; doi:10.1007/s00228-023-03476-6)

European Journal of Clinical Pharmacology

Supplementary Material for: Results From a First-in-Human Study of Dersimelagon, an Investigational Oral Selective MC1R Agonist

Akihito Ogasawara, PhD*, Kei Ogawa, MSc, Ryosuke Ide, MSc, Yuka Ikenaga, MSc , Chie Fukunaga, BSc, Satoshi Nakayama, MSc, Minoru Tsuda, PhD

*Corresponding author

Akihito Ogasawara, PhD

Mitsubishi Tanabe Pharma Corporation

1-1-1, Marunouchi Chiyoda-ku

Tokyo 100-8205, Japan

Telephone: +81-80-6236-3533

email: ogasawara.akihito@mm.mt-pharma.co.jp

Methods

PK study in rats

Male Sprague-Dawley rats (Crl:CD) (n=16) were used to assess the pharmacokinetics (PK) of dersimelagon following a single oral or intravenous (IV) dose. The dosing formulations were prepared immediately before use for oral administration at doses of 0.3, 1, and 3 mg/kg using 0.5% (w/v) methyl cellulose (Sigma-Aldrich) aqueous solution as a vehicle. The dosing formulation as 10 mL/kg volume was administered into the stomach via an oral tube using a syringe. The dosing formulations for IV administration were prepared at a dose of 2 mg/kg immediately before use using 5% (w/v) 2-hydroxypropyl-β-cyclodextrin aqueous solution as a vehicle. At each sampling point, 200-250 μL of blood were collected at 0.083, 0.25, 0.5, 1, 2, 4, 6, 8, 10, 12, and 24 hours after IV dosing and at 0.25, 0.5, 1, 2, 4, 6, 8, 10, 12, and 24 hours after oral dosing from the jugular vein using a heparin-Na–treated syringe without anesthesia. Each of the blood samples was transferred to a 2-methacryloyloxyethyl phosphoryl choline (MPC)–treated tube, cooled on ice, and then centrifuged at 12,000 revolutions per minute for 5 minutes at 4 °C to separate the plasma. Each plasma sample was immediately transferred to a new MPC tube and stored in a −80 °C

freezer until analysis. The plasma concentrations of dersimelagon were measured using the validated liquid chromatography-tandem mass spectrometry (LC/MS/MS) method.

PK study in monkeys

Male cynomolgus monkeys (*Macaca fascicularis*) aged 3 to 4 years (purpose bred, anti–B-virus antibody negative; n=4) were used to assess the PK of dersimelagon following a single oral or IV dose. The dosing formulations were prepared immediately before use for oral administration at a dose of 3 mg/kg using 0.5% (w/v) methyl cellulose (Shin-Etsu Chemical Co., Ltd) aqueous solution as a vehicle. The dosing formulation as 10 mL/kg volume was administered into the stomach via the nasal cavity using a disposable catheter and syringe. For IV administration, dosing formulation at a dose of 1 mg/kg was prepared immediately before use and employed 5% (w/v) hydroxypropyl-β-cyclodextrin solution as a vehicle. The individual dose volume was calculated based on the body weight on the dosing day. Blood was drawn from the femoral vein with a syringe and treated with an anticoagulant (EDTA-2K) at 0.083, 0.25, 0.5, 1, 2, 4, 6, 8, 24, and 48 hours after IV dosing and at 0.25, 0.5, 1, 2, 4, 6, 8, 24, and 48 hours following oral dosing. The blood was immediately transferred to an MPC polymer-treated tube and cooled on ice, and plasma was obtained by centrifugation (4 °C, 1700×*g*, 15 minutes). The obtained plasma samples were aliquoted into an MPC polymer-treated tube using an MPC polymer-treated tip and stored in a deep freezer (acceptable range: −70 °C or below). The plasma concentrations of dersimelagon were measured using the validated LC/MS/MS method.

In vitro plasma protein binding

The in vitro protein binding of [^{14}C]3131950 (dersimelagon free base) was evaluated in the plasma of rats, cynomolgus monkeys, and humans by the equilibrium dialysis method. A portion of [^{14}C]3131950 (dersimelagon free base) was serially diluted with ethanol to prepare [^{14}C]3131950 solutions of 10, 100, and 900 µg eq/mL (referred to as µg/mL). A portion of the [^{14}C]3131950 solutions was added to rat, monkey, or human plasma at 1% to give [^{14}C]3131950-spiked plasma samples (n=1 at each concentration). Immediately after preparation, a 0.5-mL aliquot of the dersimelagon free base−spiked plasma sample and a 0.75-mL aliquot of phosphate buffered saline (PBS) was transferred to the sample chamber and to the buffer chamber of the rapid equilibrium dialysis device, respectively, (n = 4 at each concentration). The device was incubated for 8 hours at 37 °C in a CO_2 incubator with

shaking. After incubation, an aliquot in the sample chamber and in the buffer chamber were measured for radioactivity. The [^{14}C]3131950 concentrations in the sample chamber and the buffer chamber were calculated from the measured radioactivity and the specific radioactivity of [^{14}C]3131950. The binding to plasma protein was calculated for each device by Microsoft Excel 2010 (Microsoft, Redmond, WA, US) using the following equation: protein binding (%) = $(1 - C_f / C_p) \times 100$, where C_f is the radioactivity concentration in the buffer chamber (dpm/mL) and C_p is the radioactivity concentration in the sample chamber (dpm/mL).

eTable 1. Study design

Part	Population	Dietary status	Formulation	Cohort	Dose	Treatment
A	White male	Fasted	Suspension	1[a]	1 mg	6 active; 2 placebo
				2[a]	3 mg[b]	6 active; 2 placebo
				3[a]	10 mg[b]	6 active; 2 placebo
				4[a]	30 mg[b]	6 active; 2 placebo
				5[a]	100 mg[b]	6 active; 2 placebo
				6[a]	300 mg[b]	6 active; 2 placebo
				7[a]	600 mg[b]	6 active; 2 placebo
B[c]	White male	Fed	Suspension	-	100 mg[b]	6 active; 2 placebo
C[c]	White male	Fed/fasted	Tablet	-	100 mg[b]	8 active
D	White female	Fasted[d]	Tablet[e]	-	100 mg[b]	6 active; 2 placebo
E[g]	Male with Fitzpatrick skin types II-IV only	Fed[d]	Suspension[e]	1	30 mg[f]	9 active; 3 placebo
			Tablet[e]	2	150 mg[f]	9 active; 3 placebo
				3	300 mg[f]	9 active; 3 placebo
	Male with Fitzpatrick skin type V or IV only[h]			4	450 mg[i]	9 active; 3 placebo
F	Black male	Fasted[d]	Tablet[e]	-	100 mg[b]	6 active; 2 placebo
G[c]	Japanese male	Fasted[d]	Tablet[e]	-	100 mg[b]	6 active; 2 placebo
H	Older male	Fasted[d]	Tablet[e]	-	100 mg[b]	6 active; 2 placebo

[a]All cohorts in Part A included two sentinel participants (one active and one placebo). The remaining six participants (five active and one placebo) were dosed at least 24 hours following the sentinel participants.

[b]Dose was confirmed based on available safety, tolerability, and pharmacokinetic (PK) data.

[c]Data not presented in this manuscript.

[d]Dietary status was confirmed following review of emerging data from Parts B and C.

[e]Formulation was selected based on review of emerging data from Parts B and/or C.

[f]Dose was confirmed based on available safety, tolerability, and PK data from Part A and preceding cohorts in Part E. Dosing frequency in Part E (once daily) was confirmed following review of PK data from Part A.

[g]Individual cohorts in Part E were conducted in a staggered approach (ie, participants within each cohort did not receive their first dose of dersimelagon/placebo on the same day).

[h]Based on emerging pigmentation data, this cohort included participants with Fitzpatrick skin type V (very rarely burns, tans very easily) or type VI (never burns, always tans) only.

[i]Available PK data from this study, including Part F, confirmed a similar PK profile between White and Black participants and predicted exposure to 450 mg to be lower than the PK stopping criteria. Dosing frequency (once daily) was also confirmed.

eTable 2. Fitzpatrick skin type of participants in the multiple ascending dose study (Part E)

Fitzpatrick skin type	Statistics	Placebo (*N*=12)	Dersimelagon				Overall (*N*=36)
			30 mg (*N*=9)	150 mg (*N*=9)	300 mg (*N*=9)	450 mg (*N*=9)	
Type II	*n* (%)	0 (0.0%)	1 (11.1%)	0 (0.0%)	0 (0.0%)	0 (0.0%)	1 (2.8%)
Type III	*n* (%)	4 (33.3%)	3 (33.3%)	8 (88.9%)	4 (44.4%)	0 (0.0%)	15 (41.7%)
Type IV	*n* (%)	5 (41.7%)	5 (55.6%)	1 (11.1%)	5 (55.6%)	0 (0.0%)	11 (30.6%)
Type V	*n* (%)	3 (25.0%)	0 (0.0%)	0 (0.0%)	0 (0.0%)	9 (100.0%)	9 (25.0%)

Note: Overall column includes all active dose levels. Percentages are based on the number of participants in each treatment group.
n, number of observations; *N*, number of participants.

eTable 3. Analysis of dose proportionality of dersimelagon (Part A and Part E)

	Day	Parameter	Slope estimate	95% CI lower	95% CI upper
**Part A (SAD) (*N*=42/*n*=42)**	-	C_{max} (ng/mL)	1.05	1.00	1.10
		$AUC_{0-\infty}$ (ng·h/mL)	1.12	1.07	1.17
**Part E (MAD) (*N*=36/*n*=36)**	Day 1	C_{max} (ng/mL)	1.12	0.98	1.27
		$AUC_{0-\infty}$ (ng·h/mL)	1.19	1.07	1.30
	Day 14[a]	C_{max} (ng/mL)	1.25	1.13	1.36
		$AUC_{0-\tau}$ (ng·h/mL)	1.26	1.14	1.39

Note: Estimates are from the power model fitted with log PK parameter as the response variable and log of dose as a fixed effect.
$AUC_{0-\infty}$, area under the plasma concentration-time curve from time zero to infinity; $AUC_{0-\tau}$, area under the plasma concentration-time curve over the dosing interval; C_{max}, maximum observed plasma concentration; *MAD*, multiple ascending dose; *N*, number of participants; *n*, number of observations; *SAD*, single ascending dose.
[a]Calculated on the basis of *n*=29 observations.

eTable 4. Pharmacokinetic parameters of dersimelagon in rats and monkeys

Species	Route of administration	Dose (mg/kg)	C_{max} (ng/mL)	T_{max} (h)	$t_{1/2}$ (h)	$AUC_{0\text{-last}}$ (ng·h/mL)	$AUC_{0\text{-}\infty}$ (ng·h/mL)	CL (mL/h/kg)	V_{ss} (mL/kg)	F (%)
**Rat**	Oral	0.3	2.5[a]	0.5[a]	NC	5.7[a]	NC	NC	NC	NC
		1	17.0[a]	0.6[a]	2.4[a]	34.5[a]	35.5[a]	NC	NC	7.2[a]
		3	42.3[a]	0.5[a]	2.0[b]	84.3[a]	102[b]	NC	NC	6.9[b]
	Intravenous	2	NC	NC	2.4[c]	969[a]	983[c]	2049[c]	1629[c]	NC
**Monkey[a]**	Oral	3	167	1.1	5.2	918	922	NC	NC	31.0
	Intravenous	1	NC	NC	5.9	953	955	1097	1930	NC

Note: Animals used include Sprague-Dawley rats and cynomolgus monkeys.
$AUC_{0\text{-}\infty}$, area under the plasma concentration-time curve from time zero to infinity; *CL*, total clearance; C_{max}, maximum observed plasma concentration; *F*, absolute bioavailability; *NC*, not calculated; $t_{½}$, apparent plasma terminal elimination half-life; T_{max}, time to maximum plasma concentration; V_{ss}, volume of distribution at steady state.
[a]Values presented as mean of four animals.
[b]Values presented as mean of two animals.
[c]Values presented as mean of three animals.

eTable 5. In vitro protein binding of dersimelagon free base in plasma of rat, cynomolgus monkey, and human

Species	Dersimelagon free base concentration (µg/mL)	Protein binding (%)
**Rat**	0.1	98.3 ± 0.1
	1	98.2 ± 0.1
	9	97.8 ± 0.1
**Cynomolgus monkey**	0.1	97.2 ± 1.3
	1	96.7 ± 3.1
	9	97.3 ± 1.4
**Human**	0.1	98.3 ± 0.3
	1	98.4 ± 0.0
	9	98.2 ± 0.1

Data represent the mean ± SD of 3 or 4 samples of pooled plasma.

eFig. 1 Dose-normalized plasma pharmacokinetic parameters of dersimelagon versus dose on a linear scale (Part A)

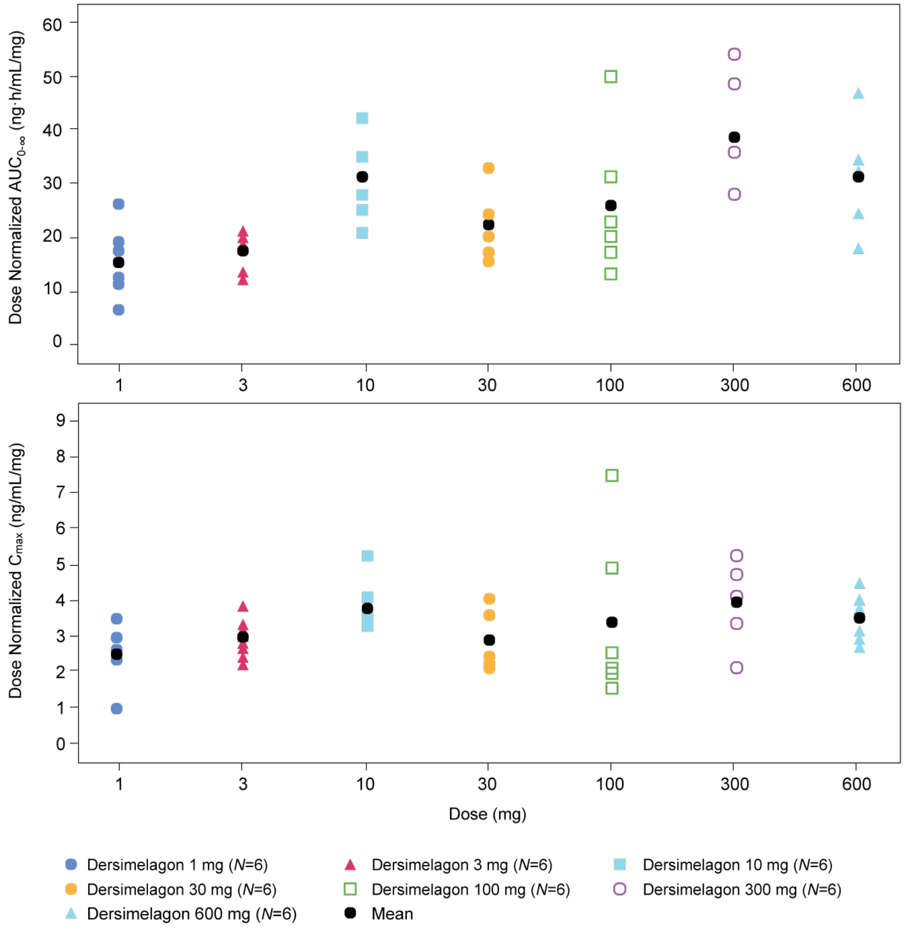

$AUC_{0\text{-}\infty}$, area under the plasma concentration-time curve from time zero to infinity; *C_{max}*, maximum observed plasma concentration.

eFig. 2 Mean plasma concentration-time curves from 0-24 hours to assess effect of sex, age, and race (semi-logarithmic scale)

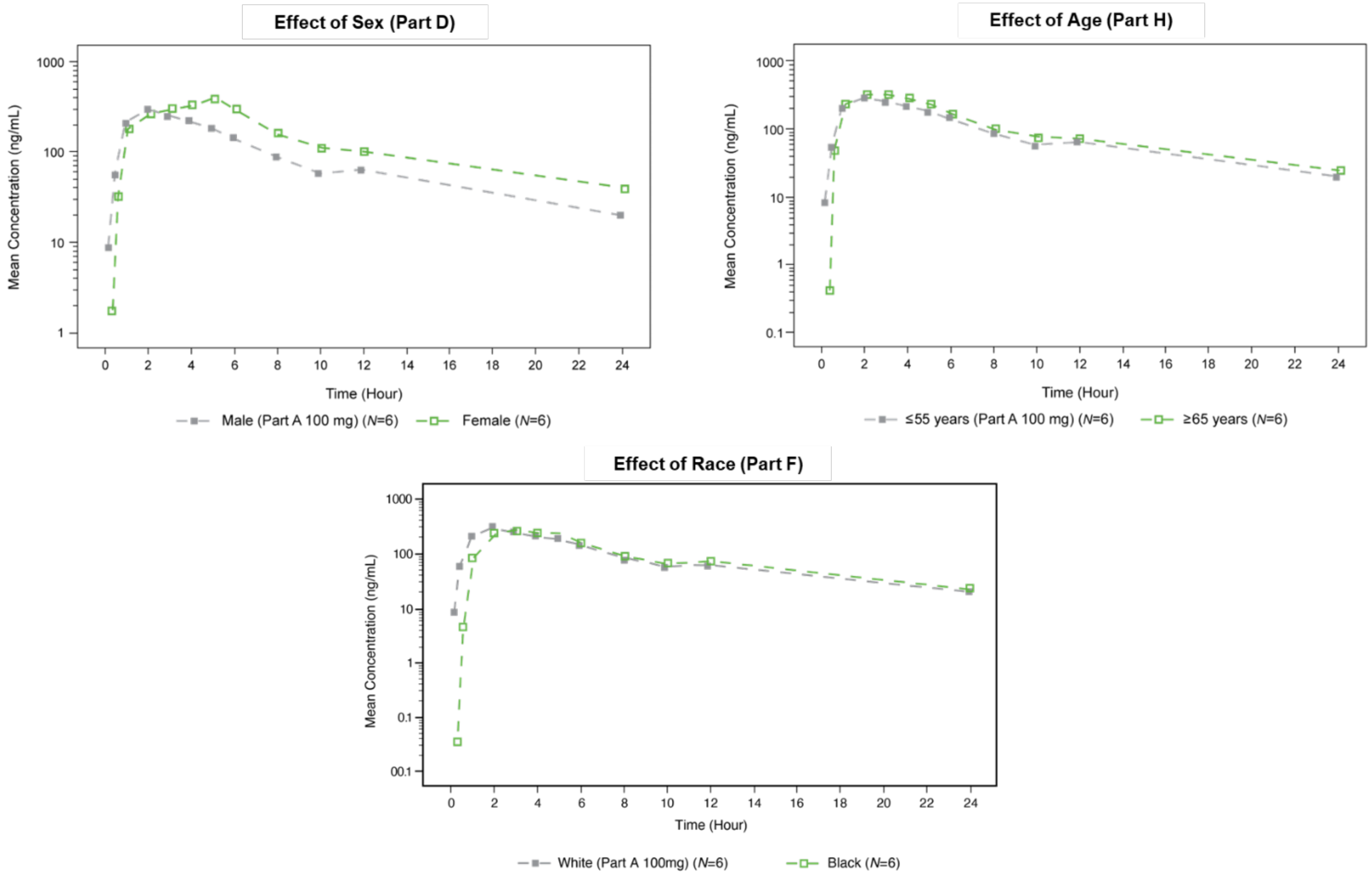

eFig. 3 Dose-normalized plasma pharmacokinetic parameters of dersimelagon versus dose on a linear scale (Part E)

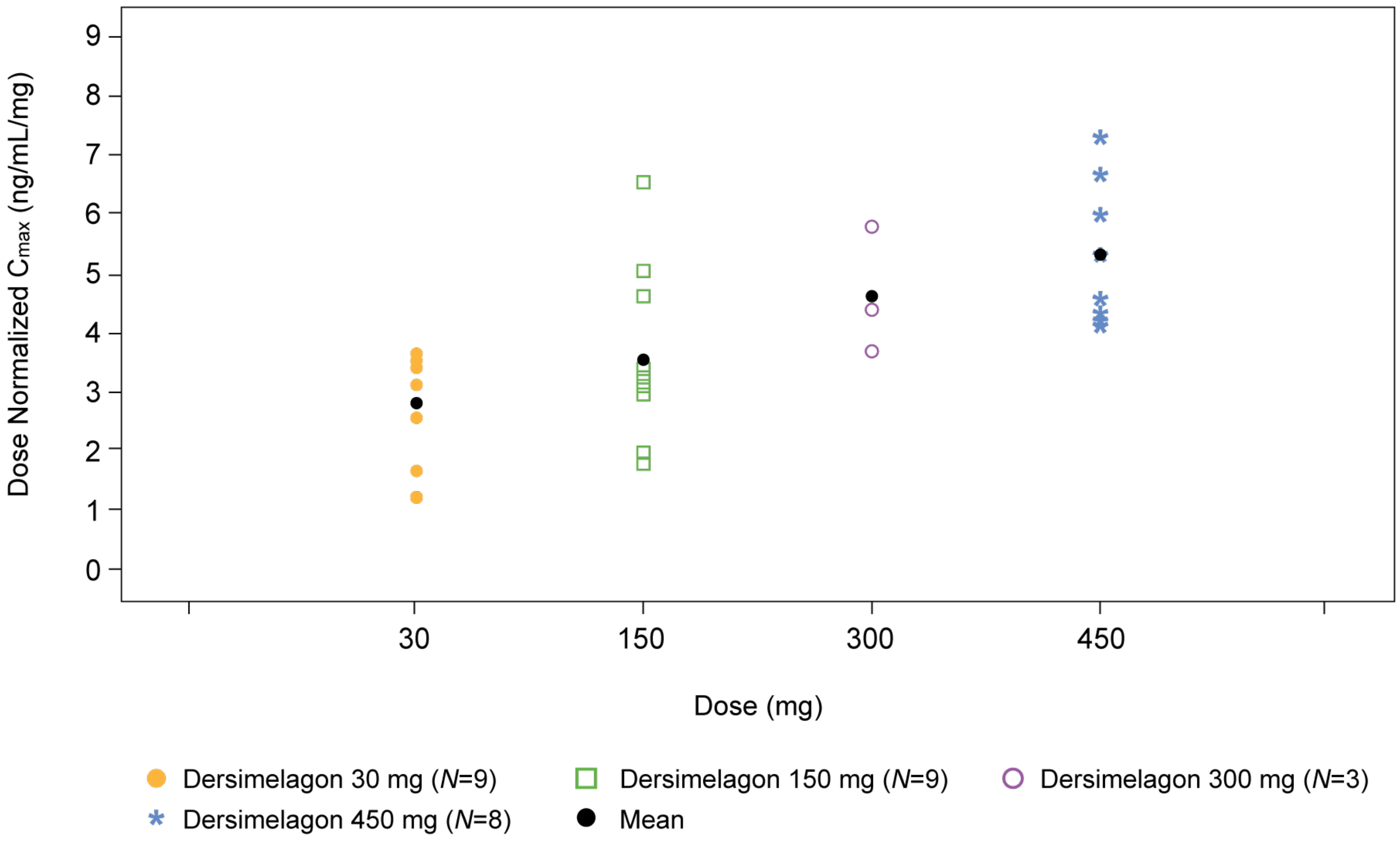

C_{max}, maximum observed plasma concentration.

eFig. 4 Mean trough concentration-time curves from day 1 to day 14 (Part E)

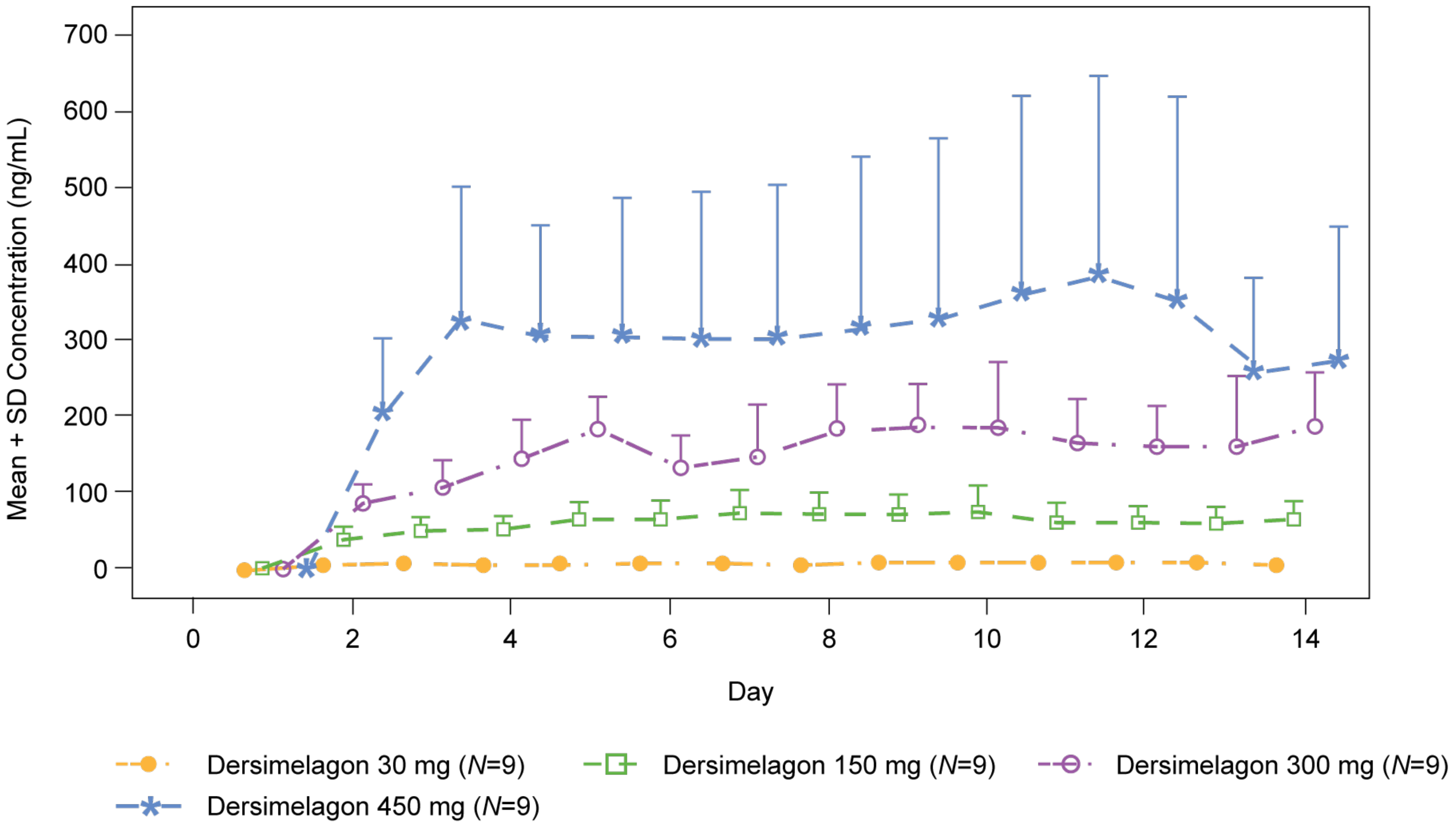

Supplement: Supplementary file 1 — Supplementary file1 (PDF 550 KB) [file 228_2023_3476_MOESM1_ESM.pdf]
